# Stochastic Analysis of the Efficiency of a Wireless Power Transfer System Subject to Antenna Variability and Position Uncertainties

**DOI:** 10.3390/s16071100

**Published:** 2016-07-19

**Authors:** Marco Rossi, Gert-Jan Stockman, Hendrik Rogier, Dries Vande Ginste

**Affiliations:** IBCN/Electromagnetics Group, Department of Information Technology, Ghent University/iMinds, Technologiepark Zwijnaarde 15, Ghent B-9052, Belgium; gertjan.stockman@intec.ugent.be (G.-J.S.); hendrik.rogier@intec.ugent.be (H.R.); dries.vande.ginste@intec.ugent.be (D.V.G.)

**Keywords:** wireless power transfer, power transfer efficiency, textile antenna, polynomial chaos, stochastic collocation, stochastic testing, uncertainty quantification, radiative near-field

## Abstract

The efficiency of a wireless power transfer (WPT) system in the radiative near-field is inevitably affected by the variability in the design parameters of the deployed antennas and by uncertainties in their mutual position. Therefore, we propose a stochastic analysis that combines the generalized polynomial chaos (gPC) theory with an efficient model for the interaction between devices in the radiative near-field. This framework enables us to investigate the impact of random effects on the power transfer efficiency (PTE) of a WPT system. More specifically, the WPT system under study consists of a transmitting horn antenna and a receiving textile antenna operating in the Industrial, Scientific and Medical (ISM) band at 2.45 GHz. First, we model the impact of the textile antenna’s variability on the WPT system. Next, we include the position uncertainties of the antennas in the analysis in order to quantify the overall variations in the PTE. The analysis is carried out by means of polynomial-chaos-based macromodels, whereas a Monte Carlo simulation validates the complete technique. It is shown that the proposed approach is very accurate, more flexible and more efficient than a straightforward Monte Carlo analysis, with demonstrated speedup factors up to 2500.

## 1. Introduction

With the advent of the Internet of Things (IoT), radio frequency identification (RFID) systems and, in general, radio frequency (RF) sensors and actuators have acquired significant importance. Distributed in our surroundings in a pervasive and inconspicuous way, these elementary components interact with each other to collect, process and exchange data [1,2]. The potential applications leveraged by the IoT paradigm are manifold and range from transportation over logistics to healthcare and from infrastructure monitoring to emergency services. As a result, assessing the correct operation of the systems involved is of paramount importance.

One of the main requirements of the IoT is that RFID components and RF sensors need to be small, low cost and not limited in lifetime by the duration of a battery [1]. Therefore, they are often designed to be passive and they rely on wireless power transfer (WPT) for their activation and operation [3,4]. Up to now, WPT in the reactive near-field and in the far-field has been widely investigated in literature [5,6,7,8]. However, the proposed solutions can be considered sub-optimal for several reasons. On the one hand, WPT operating in the reactive near-field can achieve high power transfer efficiency (PTE), but it requires source and target to be very close to each other [9]. Moreover, variations in distance between the devices strongly affect the resonance frequency and the load impedance of the system. On the other hand, the PTE achieved by far-field schemes is often too low. As a result, research efforts have recently shifted to WPT in the radiative near-field (Fresnel region), where the PTE, although rapidly decreasing with distance, can still be high enough to power sensor networks [10,11,12].

The most effective way to assess the performance of a WPT system is to study its transfer efficiency, since random fluctuations may alter the incoming power at the receiving element, whereas the output voltage level delivered by the power management system is typically first stabilized by a voltage regulator to provide a constant supply voltage needed to feed electronic circuits. To this end, several numerical techniques may be leveraged, such as source reconstruction methods [13], multipole expansion of the electromagnetic field [14], model reduction combined with full-wave solvers [15] and domain decomposition methods [16]. A very efficient approach has recently been proposed in [17,18], where the electromagnetic interaction among arbitrarily positioned radiating devices is modeled by means of their measured or simulated radiation patterns, allowing for device repositioning without requiring new simulations or measurements. Moreover, no constraint is enforced on the antenna configurations considered, as long as their radiation pattern is provided.

Although this approach efficiently quantifies the PTE of a WPT system for different positions and rotations of the antennas, it cannot assess how the PTE varies for a given system setup when devices undergo small random rotations or when their mutual position is affected by uncertainties. Moreover, the method is based on a single simulation of the radiation pattern of each element of the system, which typically corresponds to its nominal design. However, the radiation characteristics of the actually deployed antennas deviate from the nominal ones due to the inevitable uncertainties on the design parameters arising during the production process. As a result, the antenna variability has to be included in the WPT model to correctly estimate the PTE of the system.

In this manuscript, we propose a stochastic collocation method (SCM) based approach, leveraging on gPC expansions [19,20]. The formalism overcomes the limitations mentioned in the previous paragraph as it efficiently allows assessing the impact of position uncertainties and antenna variability on the PTE of a WPT system. More specifically, we start from given probability density functions (PDFs) according to which the antennas’ design parameters vary. Next, we introduce a generalized polynomial chaos (gPC) expansion for each antenna configuration to model the corresponding variations in its radiation characteristics. Furthermore, on a higher level, a second gPC expansion assesses the impact of both antenna variability and position uncertainties on the PTE of the system. In this way, the analysis of the WPT system requires a lower number of simulations compared to a single gPC expansion accounting for all variations.

The proposed approach is demonstrated on a simple WPT system consisting of a transmitting horn antenna and a receiving Industrial, Scientific and Medical (ISM) textile antenna operating at 2.45 GHz, which is connected to a rectifier circuit to deliver direct current (DC) power to a load [18]. For this receiving antenna, we use experimentally determined probability density functions of the design parameters [21,22]. The results are found to be as accurate as those obtained by the traditional Monte Carlo method, here used to validate our technique. However, owing to the considerably lower number of simulations needed to construct a gPC expansion compared to the number required for Monte Carlo method to converge, the proposed approach proves to be much more efficient and flexible. The polynomial chaos expansion has been applied to model lumped circuits and distributed interconnects [23,24], multiport systems [25], the effect of geometric and material variations in scattering problems [26,27], in Direction of Arrival (DOA) estimation [28] and in antenna design [21,22]. However, to our best knowledge, the application of uncertainty quantification to a WPT system is completely new.

This manuscript is organized as follows. In Section 2, we briefly describe both the WPT model [17] and the SCM. Then, in Section 3, the results for a WPT consisting of a transmitting horn antenna and a receiving ISM textile antenna connected to a rectifier circuit are presented and then discussed in Section 4. Conclusions are summarized in Section 5.

Notations: We denote field vectors by underlined letters, e.g., $\underline{v}$, and unit vectors with a “hat”, e.g., $\hat{\underline{v}}$. All sources and fields are assumed to be time harmonic with angular frequency *ω* and time dependencies $e^{j\omega t}$ are suppressed. Vector elements and arrays are represented by boldface characters, e.g., x. For a given array x∈C, $x^{T}$ denotes its transpose, whereas ∥x∥ denotes its Euclidean norm.

## 2. Materials and Methods

### 2.1. Wireless Power Transfer System Model

**Figure 1:**
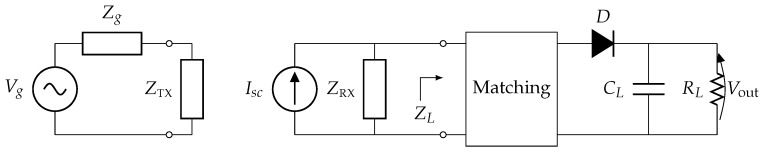
Equivalent circuit of a wireless power transfer (WPT) link, i.e., a transmit antenna and a receive antenna with rectifier (rectenna).

Consider a simple WPT system consisting of a transmitting antenna TX and a receiving antenna RX. We assume, for simplicity, that both TX and RX are one-port devices with radiation impedances $Z_{\mathrm{TX}}$ and $Z_{\mathrm{RX}}$, respectively, and we represent them by means of two equivalent circuits [29] as in Figure 1. More specifically, the transmitter is driven by means of a Thévenin generator composed of a sinusoidal voltage source $V_{g}$ and an internal impedance $Z_{g}$, whereas the receiving antenna is modeled as a Norton equivalent with a short circuit current $I_{sc}$ and load impedance $Z_{L}$. The receiver also includes a matching circuit and a rectifier to transform the alternating current (AC) power received by the antenna into the direct current (DC) power delivered to the load $R_{L}$.

The short circuit current $I_{sc}$ is computed as:

(1) $$\begin{array}{c} \begin{array}{c} I_{sc}=-\frac{1}{V_{0}}\int_{V_{RX}}{\underline{e}}^{inc}\left(\underline{r}\right)\cdot \underline{j}\left(\underline{r}\right)d\underline{r} \end{array} \end{array}$$

where $V_{0}$ is a pertinent normalization factor [30], $V_{RX}$ is the volume of RX, ${\underline{e}}^{inc}\left(\underline{r}\right)$ is the electric field incident on the receiver, $\underline{j}\left(\underline{r}\right)$ is the current density impressed on in $V_{RX}$ and $\underline{r}$ is the position vector. It was shown in [17] that, in the radiative near-field, $I_{sc}$ can be expressed as follows:

(2) $$\begin{array}{c} \begin{array}{c} I_{sc}=-\frac{1}{ZV_{0}}\int_{\Omega }\;T\left({\underline{r}}_{\mathrm{TX},\mathrm{RX}},\hat{\underline{k}}\right){\underline{F}}_{\mathrm{TX}}\left(\hat{\underline{k}}\right)\cdot {\underline{F}}_{\mathrm{RX}}\left(-\hat{\underline{k}}\right)\;d\hat{\underline{k}} \end{array} \end{array}$$

where we integrate over the Ewald sphere Ω. Furthermore, ${\underline{F}}_{\mathrm{TX}}\left(\hat{\underline{k}}\right)$ and ${\underline{F}}_{\mathrm{RX}}\left(\hat{\underline{k}}\right)$ are the radiation patterns of the transmitter and the receiver, respectively, $Z=\frac{\mu }{\epsilon }$ is the wave impedance of the background medium, $\hat{\underline{k}}=\sin\theta \cos\phi \hat{\underline{x}}+\sin\theta \sin\phi \hat{\underline{y}}+\cos\theta \hat{\underline{z}}$ is the wave vector in spherical coordinates, $T({\underline{r}}_{\mathrm{TX},\mathrm{RX}},\hat{\underline{k}})$ is a translation operator that allows efficient translations between the two antennas and ${\underline{r}}_{\mathrm{TX},\mathrm{RX}}$ is the relative position between the phase centers of the two devices. The operator $T({\underline{r}}_{\mathrm{TX},\mathrm{RX}},\hat{\underline{k}})$ is calculated as follows:

(3) $$\begin{array}{c} \begin{array}{c} T\left({\underline{r}}_{\mathrm{TX},\mathrm{RX}},\hat{\underline{k}}\right)\approx \sum_{l=0}^{L}\left(2l+1\right)j^{-l}h_{l}^{\left(2\right)}\left(k\right|{\underline{r}}_{\mathrm{TX},\mathrm{RX}}\left|\right)P_{l}\left(\hat{\underline{k}}\cdot {\hat{\underline{r}}}_{\mathrm{TX},\mathrm{RX}}\right) \end{array} \end{array}$$

where *j* is the imaginary unit, $h_{l}^{\left(2\right)}\left(\cdot \right)$ is the *l*-th order spherical Hankel function of the second kind, and $P_{l}\left(\cdot \right)$ is the Legendre polynomial of degree *l*. The number *L* determines the accuracy of the approximation in Equation (3) and traditional guidelines are followed to select it [31]. The relation in Equation (2) is valid only as long as the two antennas are not positioned in each other’s reactive near-field, thus with $|{\underline{r}}_{\mathrm{TX},\mathrm{RX}}|$ at least equal to a sixth of the wavelength.

**Figure 2:**
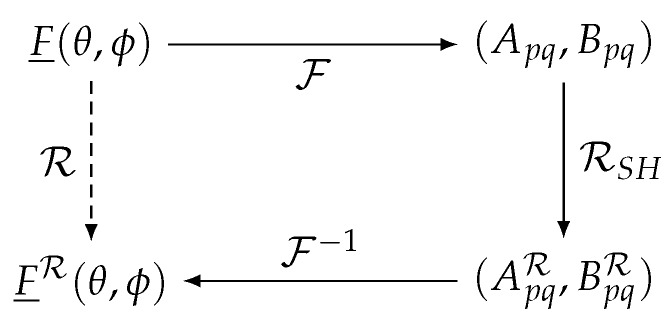
Rotation of $\underline{F}\left(\theta ,\phi \right)$ to ${\underline{F}}^{R}\left(\theta ,\phi \right)$ using the spherical harmonics domain.

The rotation of the devices around their phase center is included in the described formalism by applying the appropriate rotation R to the radiation patterns ${\underline{F}}_{\mathrm{TX}}\left(\hat{\underline{k}}\right)$ and ${\underline{F}}_{\mathrm{RX}}\left(\hat{\underline{k}}\right)$ in the spherical harmonics domain, as shown in Figure 2. Thereto, first, the radiation pattern $\underline{F}\left(\hat{\underline{k}}\right)=F_{\theta }\left(\hat{\underline{k}}\right)\hat{\underline{\theta }}+F_{\phi }\left(\hat{\underline{k}}\right)\hat{\underline{\phi }}$ is expanded into spherical harmonics $A_{pq}$ and $B_{pq}$ by means of the transformation F, given by [32]:

(4) $$\begin{array}{c} \begin{array}{c} \left\{\begin{array}{c} A_{pq}\\ B_{pq} \end{array}\right\}=-\frac{1}{p(p+1)}\int_{\phi =0}^{2\pi }\int_{\theta =0}^{\pi }\;\left[q\left\{\begin{array}{c} jF_{\phi }\left(\theta ,\phi \right)\\ -F_{\theta }\left(\theta ,\phi \right) \end{array}\right\}Y_{pq}^{*}\left(\theta ,\phi \right)+\sin\theta \left\{\begin{array}{c} -F_{\theta }\left(\theta ,\phi \right)\\ jF_{\phi }\left(\theta ,\phi \right) \end{array}\right\}\frac{dY_{pq}^{*}\left(\theta ,\phi \right)}{d\theta }\right]\;d\theta d\phi \end{array} \end{array}$$

where the unit vector $\hat{\underline{k}}$ has been replaced by (θ,ϕ), $Y_{pq}\left(\theta ,\phi \right)$ and their complex conjugates $Y_{pq}^{*}\left(\theta ,\phi \right)$ are the orthonormalized scalar spherical harmonics. Then, the coefficients $A_{pq}$ and $B_{pq}$ are rotated in the spherical harmonics domain ($R_{SH}$) by means of Wigner D-matrices. The rotated coefficients $A_{pq}^{R}$ and $B_{pq}^{R}$ are calculated as [33]

(5) $$\begin{array}{c} \begin{array}{c} \left\{\begin{array}{c} A_{pq}^{R}\\ B_{pq}^{R} \end{array}\right\}=\left\{\begin{array}{c} A_{pq}\\ B_{pq} \end{array}\right\}\sum_{|r|\leq p}e^{-jq\gamma }d_{pq}^{r}\left(\beta \right)e^{-jr\alpha } \end{array} \end{array}$$

with $d_{pq}^{r}\left(\beta \right)$ the Wigner small d-matrix, given by [34]

(6) $$\begin{array}{c} \begin{array}{c} d_{pq}^{r}\left(\beta \right)={\left(-1\right)}^{r-q}\sqrt{(p+r)!(p-r)!(p+q)!(p-q)!}\cdot \sum_{s}{\left(-1\right)}^{s}\frac{{\left(\cos\frac{\beta }{2}\right)}^{2(p-s)+q-r}{\left(\sin\frac{\beta }{2}\right)}^{2s-q+r}}{(p+q-s)!s!(r-q+s)!(p-r-s)!} \end{array} \end{array}$$

The range of *s* is determined such that all factorials are nonnegative. *α*, *β* and *γ* in Equations (5) and (6) are the standard Euler angles that define the rotation using the *z*-*y*-*z* convention in a right-handed frame. The angles (*α*, *β*, *γ*) are related to the desired inclination and azimuthal angles *θ* and *ϕ* by choosing α=ϕ, β=θ and γ=0. Finally, the rotated radiation pattern ${\underline{F}}^{R}\left(\theta ,\phi \right)$ is found from the rotated coefficients $A_{pq}^{R}$ and $B_{pq}^{R}$ by means of the transformation $F^{-1}$ given by [32]:

(7) $$\begin{array}{c} \begin{array}{c} \left\{\begin{array}{c} F_{\theta }^{R}\left(\theta ,\phi \right)\\ F_{\phi }^{R}\left(\theta ,\phi \right) \end{array}\right\}=\sum_{p=0}^{P}\sum_{|q|\leq p}\left[\left\{\begin{array}{c} A_{pq}^{R}\\ jB_{pq}^{R} \end{array}\right\}\frac{dY_{pq}\left(\theta ,\phi \right)}{d\theta }+\left\{\begin{array}{c} B_{pq}^{R}\\ jA_{pq}^{R} \end{array}\right\}\frac{qY_{pq}\left(\theta ,\phi \right)}{\sin\theta }\right] \end{array} \end{array}$$

where *P* is a parameter that sets the accuracy, which for practical reasons may be chosen equal to *L* in Equation (3) [35].

We notice from Equation (2) that only the (measured or simulated) radiation patterns of the two devices are needed to calculate the influence of the transmitter on the receiver. Moreover, the combination of the rotation mechanism of Figure 2 and the use of the translation operator $T({\underline{r}}_{\mathrm{TX},\mathrm{RX}},\hat{\underline{k}})$ in Equation (2) allow computing the short circuit current $I_{sc}$ for any set of rotations and positions of the devices. As a result, this formalism is more efficient and flexible than traditional simulations tools or measurements, which require a new computation or measurement for every rotation and repositioning.

The PTE of the WPT system considered consists of three contributions. First, we need to calculate the wireless link efficiency $\eta_{\mathrm{link}}$, which is defined as the ratio between the power $P_{\mathrm{RX}}$ delivered to the receiving antenna’s load $Z_{L}$ and the power $P_{\mathrm{TX}}$ emitted by the transmitter:

(8) $$\begin{array}{c} \begin{array}{c} \eta_{\mathrm{link}}=\frac{P_{\mathrm{RX}}}{P_{\mathrm{TX}}}=\frac{\frac{1}{2}ℜ\left(Z_{L}{\left|I_{sc}\right|}^{2}{\left|\frac{Z_{\mathrm{RX}}}{Z_{\mathrm{RX}}+Z_{L}}\right|}^{2}\right)}{\frac{1}{2Z}\int_{\Omega }\;{\left|{\underline{F}}_{\mathrm{TX}}\left(\hat{\underline{k}}\right)\right|}^{2}\;d\hat{\underline{k}}} \end{array} \end{array}$$

Then, the efficiencies $\eta_{\mathrm{match}}$ and $\eta_{\mathrm{rect}}$ of the matching and the rectifying circuits are included. Since the input impedance $Z_{L}$ of a nonlinear circuit depends on the incoming power, $\eta_{\mathrm{match}}$ has the same dependency. Therefore, we simulate it with the commercial tool Advanced Design System (ADS) by Keysight Technologies (Santa Rosa, CA, USA). As to the efficiency of the rectifier, it is calculated as $\eta_{\mathrm{rect}}=P_{\mathrm{inc}}/P_{\mathrm{DC}}$, where $P_{\mathrm{inc}}=\eta_{\mathrm{match}}\cdot P_{\mathrm{RX}}$ and $P_{\mathrm{DC}}$ are the AC power injected into the rectifier and the DC power delivered to the load $R_{L}$, respectively. The DC power $P_{\mathrm{DC}}$ is given by:

(9) $$\begin{array}{c} \begin{array}{c} P_{\mathrm{DC}}=V_{\mathrm{out}}^{2}/R_{L} \end{array} \end{array}$$

where the DC output voltage $V_{\mathrm{out}}$ is also computed by ADS. Finally, the overall PTE of the system is given by:

(10) $$\begin{array}{c} \begin{array}{c} PTE=\frac{P_{\mathrm{DC}}}{P_{\mathrm{TX}}}=\eta_{\mathrm{link}}\cdot \eta_{\mathrm{match}}\cdot \eta_{\mathrm{rect}} \end{array} \end{array}$$

### 2.2. Stochastic Collocation Method

Consider a generic system output G (such as the PTE of the WPT system) and *N* input random variables (RV) $x_{1},x_{2},\ldots ,x_{N}$ that affect it. We assume these variables to be actually independent and we collect them in the vector $x=[x_{1},x_{2},\ldots ,x_{N}]$. Then, following the Wiener-Askey scheme [19], we relate G to x by means of a polynomial chaos expansion

(11) $$\begin{array}{c} \begin{array}{c} G=f\left(x\right)=\sum_{k=0}^{K}y_{k}\varphi_{k}\left(x\right) \end{array} \end{array}$$

where $\varphi_{k}\left(x\right)$ are suitably chosen multivariate polynomial basis functions and the expansion coefficients $y_{k}$ are the unknowns to be determined. The polynomials $\varphi_{k}\left(x\right)$ are constructed to be orthonormal with respect to the PDF $P_{X}$, which describes the likelihood of the input x. Thus:

(12) $$\begin{array}{c} \begin{array}{c} <\varphi_{j}\left(x\right),\varphi_{k}\left(x\right)>=\int_{\Gamma }\varphi_{j}\left(x\right)\varphi_{k}\left(x\right)P_{X}\left(x\right)dx=\delta_{jk} \end{array} \end{array}$$

with $\delta_{jk}=0$ if i≠j, $\delta_{jk}=1$ if i=j, and Γ being the support of $P_{X}$. Consequently, $P_{X}$ acts as a weighting function. Since the considered input RVs are independent, the PDF $P_{X}$ is defined as the product of the PDFs corresponding to the single input variables. Therefore, the polynomials $\varphi_{k}\left(x\right)$ are constructed as products of *N* univariate polynomials, each one associated to a single input RV. Furthermore, the multivariate polynomials have a total degree of maximally *Q*, meaning that the sum of the orders of the univariate polynomials is at most *Q*.

Finally, by means of the Stochastic Testing (ST) algorithm [36] described in the Appendix, we select M=K collocation points $x_{m}$ and we compute both a matrix *A*, whose elements are defined as $a_{mk}=\varphi_{k}\left(x_{m}\right)$, and its inverse *B*. The coefficients $y_{k}$ in Equation (11) are then calculated as in [37]:

(13) $$\begin{array}{c} \begin{array}{c} y_{k}=\sum_{m=0}^{M}b_{mk}f\left(x_{m}\right) \end{array} \end{array}$$

where $b_{mk}$ is the mk-th element of matrix *B* and $f(x_{m})$ is the function to be approximated, evaluated in $x_{m}$.

### 2.3. Wireless Power Transfer Uncertainty Quantification

The efficiency of the WPT system under study is affected by two main issues. On the one hand, both the transmitter and the receiver may undergo uncertainties in their design parameters, since, for example, the production process yields devices that do not perfectly correspond to their nominal design. As a result, random variations may be expected on both radiation impedances $Z_{\mathrm{TX}}$ and $Z_{\mathrm{RX}}$, and on the antennas’ radiation patterns. On the other hand, the system may be conceived to operate in a given configuration and undesired small random rotations or variations in the mutual position of the antennas affect the influence of the transmitter on the receiver.

Conventionally, these problems are expected to arise simultaneously. Therefore, the variations in the WPT efficiency corresponding to both antenna variability and position uncertainies are investigated by means of gPC expansions in Equation (11), which are defined as functions of the design parameters of the antennas and of the WPT system. However, the use of a single gPC expansion to carry out the complete analysis is sub-optimal. This is understood as follows. The construction of a gPC expansion requires selecting and processing *M* collocation points $x_{m}$ to compute the coefficients $y_{k}$ in Equation (11). Even though translations and rotations are efficiently accounted for by the WPT model described in Section 2.1, both the antennas’ radiation impedances and their radiation patterns are usually computed by means of full-wave solvers, such as ADS Momentum (Keysight Technologies, Santa Rosa, CA, USA), whose simulations are typically time-consuming. As a result, accounting for the variability of the antennas requires a high number of full-wave simulations. Moreover, if the efficiency of the WPT system is evaluated for different system configurations or different position uncertainties, a new gPC expansion has to be constructed for each configuration and for each position. As a result, for each such expansion a new set of collocation points has to be selected and processed, again requiring full-wave simulations, which significantly decrease the efficiency of the method with respect to more naive approaches, such as Monte Carlo.

In order to overcome these limitations and drastically improve efficiency, the construction of the gPC expansions that model the efficiency of the whole system is preceded by an intermediate step. More specifically, for each antenna configuration undergoing variability, we introduce gPC expansions in Equation (11) of the real and the imaginary parts of both its radiation impedance *Z* and the spherical harmonic coefficients $A_{pq}$ and $B_{pq}$ in Equation (4):

(14) $$\begin{array}{ccc} Z^{re} & = & \sum_{k_{1}=0}^{K_{Z^{re}}}y_{k_{1}}\varphi_{k_{1}}\left(x^{\mathrm{VAR}}\right) \end{array}$$

(15) $$\begin{array}{ccc} Z^{im} & = & \sum_{k_{2}=0}^{K_{Z^{im}}}y_{k_{2}}\varphi_{k_{2}}\left(x^{\mathrm{VAR}}\right) \end{array}$$

(16) $$\begin{array}{ccc} A_{pq}^{re} & = & \sum_{k_{3}=0}^{K_{A_{pq}^{re}}}y_{k_{3}}\varphi_{k_{3}}\left(x^{\mathrm{VAR}}\right) \end{array}$$

(17) $$\begin{array}{ccc} A_{pq}^{im} & = & \sum_{k_{4}=0}^{K_{A_{pq}^{im}}}y_{k_{4}}\varphi_{k_{4}}\left(x^{\mathrm{VAR}}\right) \end{array}$$

(18) $$\begin{array}{ccc} B_{pq}^{re} & = & \sum_{k_{5}=0}^{K_{B_{pq}^{re}}}y_{k_{5}}\varphi_{k_{5}}\left(x^{\mathrm{VAR}}\right) \end{array}$$

(19) $$\begin{array}{ccc} B_{pq}^{im} & = & \sum_{k_{6}=0}^{K_{B_{pq}^{im}}}y_{k_{6}}\varphi_{k_{6}}\left(x^{\mathrm{VAR}}\right) \end{array}$$

where $x^{\mathrm{VAR}}$ is the vector of the antenna’s design parameters that are affected by uncertainties. These gPC expansions can now be interpreted as macromodels of the considered antennas. Once constructed, they allow accurately computing both an antenna’s radiation impedance and radiation pattern for a value of $x^{\mathrm{VAR}}$ without having to resort to full-wave simulations. After this intermediate step, we model both the link efficiency $\eta_{\mathrm{LINK}}$ and the overall PTE of the system by means of the following gPC expansions:

(20) $$\begin{array}{ccc} \eta_{\mathrm{LINK}} & = & \sum_{k_{7}=0}^{K_{\eta_{\mathrm{LINK}}}}y_{k_{7}}\varphi_{k_{7}}\left(x^{\mathrm{WPT}}\right) \end{array}$$

(21) $$\begin{array}{ccc} \text{PTE} & = & \sum_{k_{8}=0}^{K_{\text{PTE}}}y_{k_{8}}\varphi_{k_{8}}\left(x^{\mathrm{WPT}}\right) \end{array}$$

where $x^{\mathrm{WPT}}$ is the vector of all the parameters in the WPT system subject to variations, which comprises all the vectors $x^{\mathrm{VAR}}$ and the variables corresponding to position uncertainties. The procedure is now as follows. First, $M_{\eta_{\mathrm{LINK}}}$ and $M_{\mathrm{PTE}}$ collocation points $x_{m}^{\mathrm{WPT}}$ are selected to construct the gPC expansions in Equations (20) and (21), respectively. Next, for each collocation point, the gPC expansions in Equations (14)–(19) are used to rapidly compute both the radiation impedances and the radiation patterns of the antennas undergoing variability. Finally, with the calculated antennas’ radiation characteristics, the WPT model described in Section 2.1 processes the values of $x_{m}^{\mathrm{WPT}}$ corresponding to the position uncertainties in the system and computes the values of $\eta_{\mathrm{LINK}}$ and PTE in those collocation points required to calculate the coefficients $y_{k_{7}}$,$y_{k_{8}}$ in Equations (20) and (21).

The benefits of this approach are twofold. First, since for each antenna the number of design parameters undergoing variations is expected to be significantly lower than the total number of parameters affecting the system, the amount of full-wave simulations necessary to construct all antenna macromodels is expected to be substantially lower than what is required to compute a single gPC expansion that models the entire WPT system. Second, once these macromodels are available, the analysis of the WPT system can be repeated for any given system configuration at a negligible computational cost, since no full-wave simulations are required to calculate the antennas’ radiation characteristics.

## 3. Results

### 3.1. Validation Example Setup

**Figure 3:**
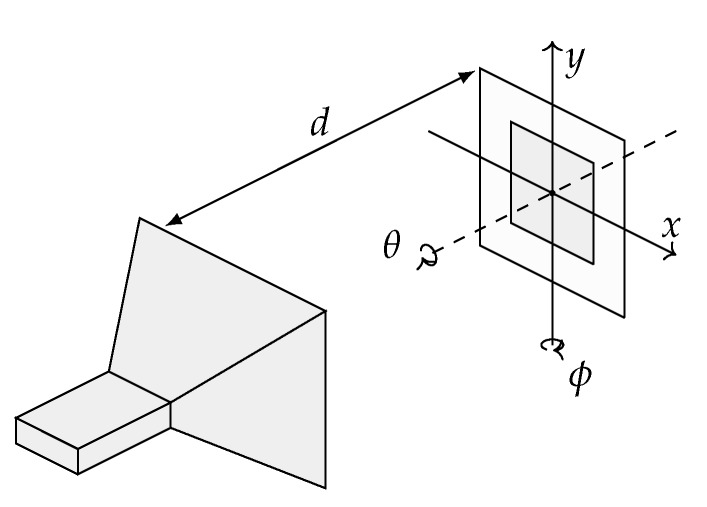
Simulation setup where an standard gain horn (SGH) acts as transmitter and a patch antenna as receiver.

**Figure 4:**
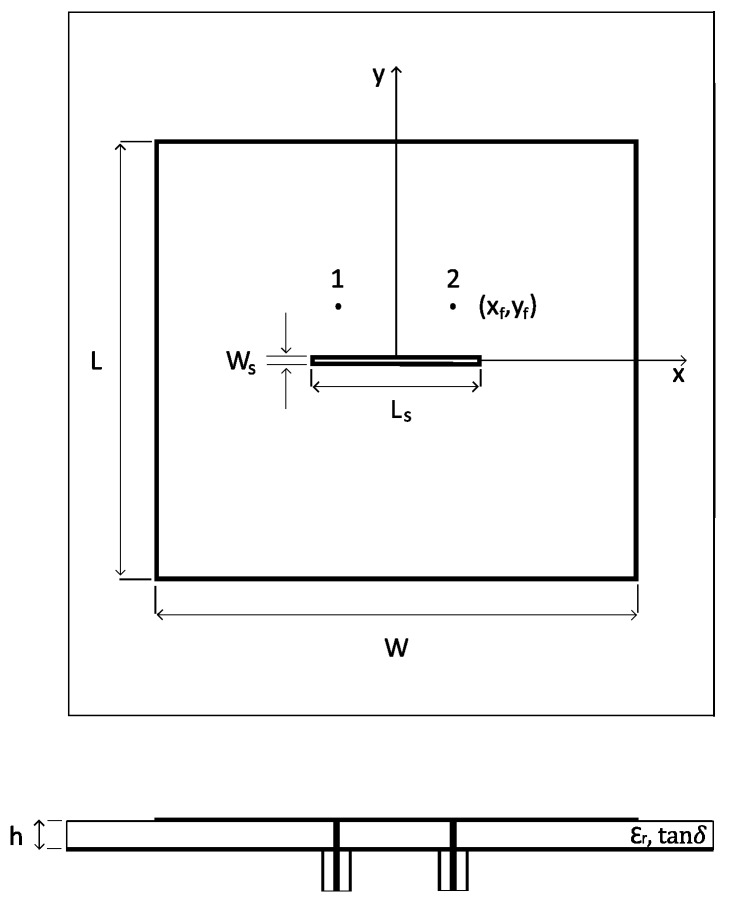
Schematic of the dual polarized probe-fed Industrial, Scientific and Medical (ISM) band textile antenna under study. (**Top panel**): Top view; (**Bottom panel**): Side view. Antenna parameters are indicated in Table 1.

The proposed approach is demonstrated on the WPT system shown in Figure 3. The transmitting device is a standard gain horn (SGH) antenna radiating at 2.45 GHz with a power of 10 dBm. The receiving device is a 2.4–2.4835 GHz ISM band textile microstrip probe-fed dual-polarized patch antenna [38], using a flexible closed-cell expanded rubber foam as substrate, and shown in Figure 4. The antennas are placed at a separation distance *d* and their phase centers are aligned.

The radiation impedance $Z_{\mathrm{TX}}$ of the SGH antenna is 50 Ω and its radiation pattern is computed by analytical expressions (see the Appendix in [17], where a=368.6 mm, b=273.1 mm, $\rho_{1}=343.9$ mm, $\rho_{2}=363.4$ mm). Instead, the 2.45 GHz ISM band textile antenna is designed and simulated by means of ADS Momentum (Keysight Technologies, Santa Rosa, CA, USA). Its nominal design parameters are reported in Table 1. Its nominal radiation impedance $Z_{\mathrm{RX}}$, which is equal for both feed 1 and feed 2, is 49.91−1.93iΩ at the frequency of 2.45 GHz. An isolation −20log|$S_{21}$| between the two feed ports of more than 15 dB is achieved within the entire ISM band.

**Figure 5:**
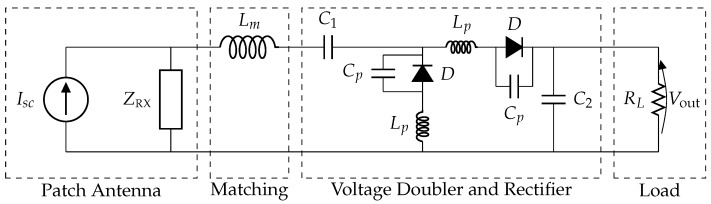
The complete schematic of a rectenna element as designed and simulated in Advanced Design System (ADS).

The wireless link efficiency $\eta_{\mathrm{link}}$ between the SGH and the textile antenna is computed by means of the formalism described in Section 2.1. The parameters *L* and *P* in Equations (3) and (7), respectively, are both set to five, leading to 36 coefficients $A_{pq}$ and $B_{pq}$. Next, a rectifier is attached to the patch antenna to form the rectenna shown in Figure 5, which is designed and simulated in ADS. More specifically, the rectenna consists of a matching network, a voltage doubler and a rectifier. The matching circuit is given by an inductor $L_{m}=5\;$nH, whereas the voltage doubler and rectifier circuit itself consist of two HSMS-2850 Schottky diodes, for which a pertinent SPICE model is used, together with their package parasitics $C_{p}=0.08\;$pF and $L_{p}=2\;$nH. The capacitors $C_{1}$ and $C_{2}\;$ are equal to 100 pF and the load resistance is equal to $R_{L}=100\;\Omega$. The matching circuit is designed to have optimal matching when $P_{\mathrm{RX}}=-10\;$dBm. However, because of the nonlinear diodes in the voltage doubler and rectifier circuit, the load impedance $Z_{L}$, and, therefore, the matching efficiency $\eta_{\mathrm{match}}$, depend on the incoming power $P_{\mathrm{RX}}$. This impedance is computed for different incoming powers by using a harmonic balance simulation in ADS and the matching efficiency is then calculated as:

(22) $$\begin{array}{c} \begin{array}{c} \eta_{\mathrm{match}}=1-{\left|\Gamma \right|}^{2}=1-{\left|\frac{Z_{L}\left(P_{\mathrm{RX}}\right)-Z_{\mathrm{RX}}^{*}}{Z_{L}\left(P_{\mathrm{RX}}\right)+Z_{\mathrm{RX}}}\right|}^{2} \end{array} \end{array}$$

Finally, the efficiency $\eta_{\mathrm{rect}}$ of the voltage doubler and rectifier is calculated as in Equation (9), and the total efficiency $\eta_{\mathrm{tot}}$ of the rectenna is given by $\eta_{\mathrm{tot}}=\eta_{\mathrm{match}}\cdot \eta_{\mathrm{rect}}$.

### 3.2. Antenna Variability

In the example under study, we only consider antenna variability of the 2.45 GHz ISM band textile antenna, since an SGH antenna is not expected to deviate from its nominal characteristic. Textile antennas, however, usually exhibit variations in their design parameters, which significantly alter their figures of merit. On the one hand, the production process inevitably introduces uncertainties in the geometrical dimensions of the antenna. On the other hand, the flexible closed-cell expanded rubber protective foam used as antenna substrate is a very non-uniform material. As a result, the value of its electrical permittivity $\epsilon_{r}$ may be quite different from the nominal one.

In [21], it is shown that variations in the patch length *L*, the patch width *W* and the electrical permittivity $\epsilon_{r}$ of the antenna have a significant impact on its radiation impedance $Z_{\mathrm{RX}}$, whereas the influence of other parameters is negligible. Moreover, a quick sensitivity analysis confirms that only variations in *L*, *W* and $\epsilon_{r}$ affect the radiation pattern of the antenna. Therefore, we relate both the real and the imaginary part $Z_{\mathrm{RX}}^{re}$ and $Z_{\mathrm{RX}}^{im}$, respectively, of the radiation impedance $Z_{\mathrm{RX}}$ of the receiving antenna, as well as the real and the imaginary part of the coefficients $A_{pq}$ and $B_{pq}$ in Equation (4), to the parameters *L*, *W* and $\epsilon_{r}$ by means of the gPC expansions in Equations (14)–(19) where thus, $x^{\mathrm{VAR}}=\left[L,W,\epsilon_{r}\right]$. As we know from [21,22] that these parameters are independent and vary according to Gaussian distributions, according to the Wiener-Askey scheme [19], the multivariate polynomials $\varphi_{k_{1}}\left(x^{\mathrm{VAR}}\right)$, $\varphi_{k_{2}}\left(x^{\mathrm{VAR}}\right)$, $\varphi_{k_{3}}\left(x^{\mathrm{VAR}}\right)$, $\varphi_{k_{4}}\left(x^{\mathrm{VAR}}\right)$, $\varphi_{k_{5}}\left(x^{\mathrm{VAR}}\right)$, $\varphi_{k_{6}}\left(x^{\mathrm{VAR}}\right)$ in Equations (14)–(19) consist of products of Hermite polynomials. Finally, the PDF $P_{X}$ in Equation (12) is given by the product of three univariate Gaussian distributions. Its mean vector ***μ*** and covariance matrix **Σ** are given by:

(23) $$\begin{array}{cc} \mu =\left[\begin{array}{c} \mu_{L}\\ \mu_{W}\\ \mu_{\epsilon_{r}} \end{array}\right]= & \left[\begin{array}{c} 45.3854\\ 44.5146\\ 1.5259 \end{array}\right]\;\begin{array}{c} \; \end{array}\text{(mm)} \end{array}$$

(24) $$\begin{array}{cc} \Sigma =\left[\begin{array}{ccc} \sigma_{L}^{2} & \sigma_{L}\sigma_{W}\rho_{LW} & \sigma_{L}\sigma_{\epsilon_{r}}\rho_{L\epsilon_{r}}\\ \sigma_{W}\sigma_{L}\rho_{LW} & \sigma_{W}^{2} & \sigma_{W}\sigma_{\epsilon_{r}}\rho_{W\epsilon_{r}}\\ \sigma_{\epsilon_{r}}\sigma_{L}\rho_{L\epsilon_{r}} & \sigma_{\epsilon_{r}}\sigma_{W}\rho_{W\epsilon_{r}} & \sigma_{\epsilon_{r}}^{2} \end{array}\right]= & \left[\begin{array}{ccc} 0.01607 & 0 & 0\\ 0 & 0.0265 & 0\\ 0 & 0 & 0.001017 \end{array}\right]\text{(mm}{}^{2}) \end{array}$$

where $\sigma_{L}$, $\sigma_{W}$ and $\sigma_{\epsilon_{r}}$ are the standard deviations of *L*, *W* and $\epsilon_{r}$, respectively, whereas $\rho_{LW}=\rho_{L\epsilon_{r}}=\rho_{W\epsilon_{r}}=0$ indicates that there is no correlation between the design parameters.

The gPC expansions of $Z_{\mathrm{RX}}^{re}$ and $Z_{\mathrm{RX}}^{im}$ are found to converge for orders of expansion with a total degree $Q_{Z_{\mathrm{RX}}^{re}}$ and $Q_{Z_{\mathrm{RX}}^{im}}$ equal to 2 and 3, respectively. In contrast, the real and the imaginary part of the coefficients $A_{pq}$ and $B_{pq}$ exhibit a more intricate behavior. Therefore, a total degree $Q_{A_{pq}}=Q_{B_{pq}}=6$ is necessary to accurately catch the variations determined by *L*, *W* and $\epsilon_{r}$ on these parameters. Since both the radiation impedance $Z_{\mathrm{RX}}$ and the radiation pattern of the antenna are computed during the same full-wave simulation, the total degree of all gPC expansions in Equations (14)–(19) is set to $Q_{\mathrm{VAR}}=6$. In this way, only one set of collocation points is selected and simulated in ADS to compute the coefficients $y_{k_{1}},y_{k_{2}},y_{k_{3}},y_{k_{4}},y_{k_{5}},y_{k_{6}}$ in Equations (14)–(19) and to construct all the gPC expansions, which is clearly beneficial in terms of computation time. In this case, a number $M_{\mathrm{VAR}}=84$ of collocation points $x_{m}^{\mathrm{VAR}}$ has been selected by means of the ST algorithm and simulated in ADS.

### 3.3. Position Uncertainties

Nominally, the WPT system shown in Figure 3 is constructed to operate in the radiative near-field with a distance *d* between the two antennas equal to 0.6m≈5λ and with their phase centers aligned at coordinates x=y=0. Furthermore, the radiating surface of the SGH antenna and the patch antenna are aligned with the xy plane, which means that the rotation angles *θ* and *ϕ* are both equal to 0${}^{\circ }$. In practice, the antennas are perturbed by small random rotations and variations in their mutual position. For the sake of conciseness, the SGH antenna is stationary and all the variations are cumulated in the position and the rotation of the 2.45 GHz ISM band antenna. Finally, since no experimental data are available to estimate the PDFs corresponding to the considered parameters, we suppose that *d*, *x*, *y*, *θ* and *ϕ* are independent and vary according to Gaussian distributions, whose mean values and standard deviations are reported in Table 2.

More specifically, we assume that the variations in the position of the ISM patch antenna in the xy plane are limited to intervals x=±2 cm and y=±2 cm, which correspond to a displacement of about half the width *W* and the length *L* of the patch. As for *d*, *θ* and *ϕ*, the variations are assumed to be large enough to account for a potential displacement in a real scenario.

### 3.4. Wireless Power Transfer Efficiency

The two gPC expansions in Equations (20) and (21) are introduced to relate both the link efficiency $\eta_{\mathrm{LINK}}$ and the overall PTE to the parameters *L*, *W*, $\epsilon_{r}$, *d*, *x*, *y*, *θ* and *ϕ*. All parameters are independent and vary according to Gaussian distributions. As a result, the multivariate polynomials $\varphi_{k_{7}}\left(x^{\mathrm{WPT}}\right)$, $\varphi_{k_{8}}\left(x^{\mathrm{WPT}}\right)$ of both the gPC expansions are Hermite polynomials, as in Section 3.2. Note, however, that upon the availability of other experimental data, other distributions for these parameters can equally be dealt with by means of the advocated gPC-based approach. We find that both expansions converge for an order of expansion corresponding to a total degree $Q_{\eta_{\mathrm{LINK}}}$ and $Q_{\mathrm{PTE}}$ equal to 4. A number $M_{\eta_{\mathrm{LINK}}}=M_{\mathrm{PTE}}=495$ of collocation points $x_{m}^{\mathrm{LINK}}$ is selected by means of the ST algorithm and processed with the WPT model and the antenna macromodels in order to compute the coefficients $y_{k_{7}}$, $y_{k_{8}}$ in Equations (20) and (21).

**Figure 6:**
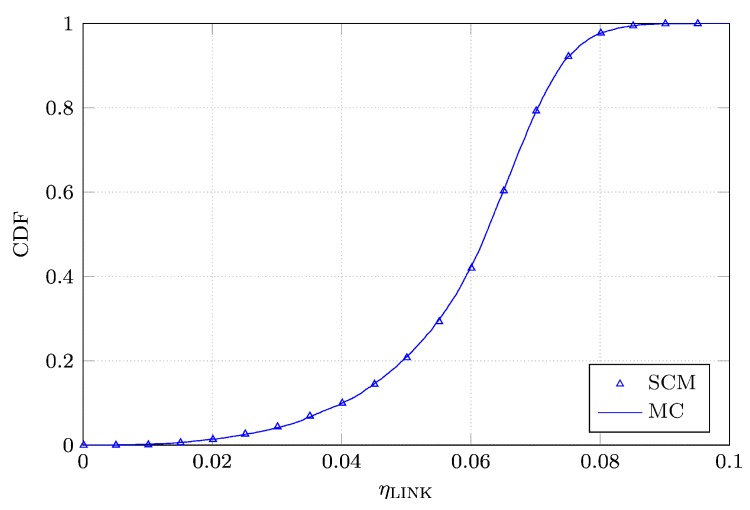
Comparison between the Cumulative Distribution Functions (CDFs) of $\eta_{\mathrm{LINK}}$ constructed with the advocated Stochastic Collocation Method (SCM) and the Monte Carlo (MC) simulations.

**Figure 7:**
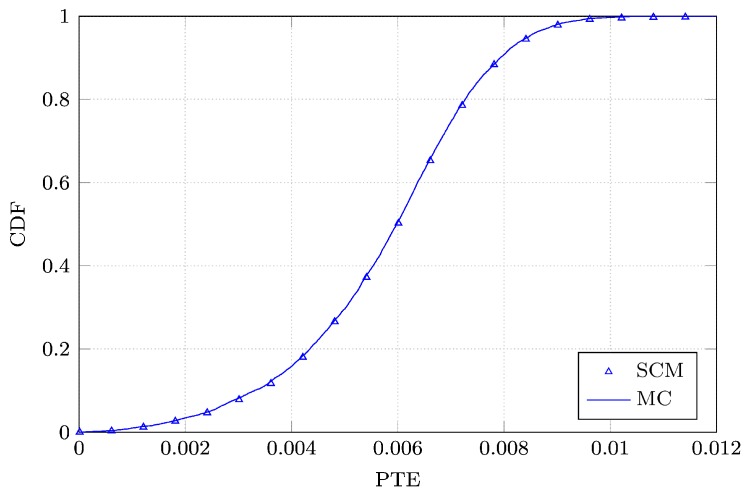
Comparison between the CDFs of the power transfer efficiency (PTE) of the WPT system constructed with the advocated SCM and the MC simulations.

Next, we perform a Monte Carlo analysis of both $\eta_{\mathrm{LINK}}$ and PTE by processing a sample set of 10,000 realizations of *L*, *W*, $\epsilon_{r}$, *d*, *x*, *y*, *θ* and *ϕ*, drawn according to their pertinent PDFs. Then, we compute the cumulative distribution functions (CDFs) of $\eta_{\mathrm{LINK}}$ and PTE based on both the Monte Carlo simulation and the SCM analysis. The curves are shown in Figure 6 and Figure 7. We notice that the CDFs are perfectly overlapping. Finally, in order to validate our analysis, we apply the Kolmogorov-Smirnov test to verify whether the CDFs of $\eta_{\mathrm{LINK}}$ and PTE and those found by means of the SCM analysis correspond to the same distribution. In particular, the maximum distance *D* between them is compared to a threshold distance $D_{\alpha }$. For $D<D_{\alpha }$, the Kolmogorov–Smirnoff test accepts the null hypothesis that both the sample sets correspond to the same distribution, with a significance level *α*. If we set the significance level *α* to 0.05, $D_{\alpha }$ equals 0.019233. The computed values of $D_{\eta_{\mathrm{LINK}}}$ and $D_{PTE}$ are equal to 0.0067 and 0.0085, respectively. Therefore, the null hypothesis of equality between the CDFs is validated with a significance level of 5%.

## 4. Discussion

The proposed approach allows quantifying the variations of both the link efficiency $\eta_{\mathrm{LINK}}$ and the PTE of a WPT system in a more efficient and flexible way than both an SCM analysis based on a single gPC expansion and a Monte Carlo analysis. As shown in Table 3, both the number of full-wave simulations and the overall CPU time necessary to perform the analysis are greatly reduced for both $\eta_{\mathrm{LINK}}$ and PTE:

More specifically, the simulation of a single realization of the 2.45 GHz ISM band antenna in ADS Momentum (Keysight Technologies, Santa Rosa, CA, USA) requires about 15 s, whereas the construction of the gPC expansions that model its radiation impedance $Z_{\mathrm{RX}}$ and the coefficients $A_{pq}$ and $B_{pq}$ takes 3.72 s, once the $M_{\mathrm{VAR}}=84$ antenna realizations have been simulated in ADS. As a result, the construction of the gPC-based macromodels for $Z_{\mathrm{RX}}$ and the radiation pattern of the antenna requires about 21 min. Then, $M_{\eta_{\mathrm{LINK}}}=M_{\mathrm{PTE}}=495$ collocation points have to be processed to construct the gPC expansions of both $\eta_{\mathrm{LINK}}$ and PTE. In order to obtain 495 samples of the link efficiency $\eta_{\mathrm{LINK}}$ by using the constructed macromodel of the antenna and the WPT model of Section 2.1, about 25 s are required. In contrast, about 32 s are necessary to collect 495 samples of the overall PTE, which include the simulations of the rectifier. Finally, the construction of both the gPC expansions of $\eta_{\mathrm{LINK}}$ and PTE takes about 25 s. Therefore, a first complete analysis of the WPT system requires about 22 min. Once the antenna characteristics have already been modeled, additional analyses of other WPT systems using this antenna or for other distributions of the position parameters take only about 1 min. In comparison, an analysis based on a single gPC, which requires direct simulation of 495 antenna realizations in ADS, takes more than 2 h. Moreover, this operation has to be repeated each time any difference is introduced in the distributions according to which the parameters of the WPT system vary. As for the Monte Carlo procedure, the simulation of 10,000 realizations by means of ADS and the WPT model of Section 2.1 requires more than 41 h. As a result, the proposed approach greatly outperforms both the Monte Carlo technique and an SCM analysis based on a single gPC expansion.

## 5. Conclusions

In this manuscript, an SCM analysis of the efficiency of a WPT system in the radiative near-field subject to antenna variability and position uncertainties has been presented. More specifically, a first SCM analysis is carried out to account for the impact of uncertainties in the design parameters of the antennas on their radiation characteristics. The resulting gPC expansions are used as macromodels that allow computing the antennas’ radiation characteristics in a more efficient way than full-wave solvers. Then, a second SCM analysis quantifies the impact of both the variability of the deployed antennas and the uncertainties in their mutual position on the efficiency of the WPT system. This is done by leveraging the previously computed gPC-based macromodels and a very efficient model for WPT systems in the radiative near-field. Finally, the proposed approach is validated by means of a WPT system consisting of an SGH antenna and a 2.45 GHz ISM band textile antenna. Compared to an SCM analysis based on a single gPC expansion, as well as to a standard MC analysis, the method shows excellent agreement and superior efficiency.

## Figures and Tables

**Table 1:** Nominal values of the antenna parameters (Figure 4).

Parameter	Nominal Value
patch length *L*	44.46 mm
patch width *W*	45.32 mm
slot length $L_{s}$	14.88 mm
slot width $W_{s}$	1 mm
feed points ($\pm x_{f},\;y_{f}$)	(±5.7, 5.7) mm
substrate height *h*	3.94 mm
permittivity $\epsilon_{r}$	1.53
loss tangent tan*δ*	0.012

**Table 2:** Mean values and standard deviations of the geometrical parameters of the link (Figure 3).

Parameter	Mean Value *μ*	Standard Deviation *σ*	3σ
*d*	0.6 m	0.01666 m	0.05 m
*x*	0 m	0.00666 m	0.02 m
*y*	0 m	0.00666 m	0.02 m
*θ*	0${}^{\circ }$	10${}^{\circ }$	30${}^{\circ }$
*ϕ*	0${}^{\circ }$	10${}^{\circ }$	30${}^{\circ }$

**Table 3:** Simulation data for the analysis of the $\eta_{\mathrm{LINK}}$ and the power transfer efficiency (PTE) of the wireless power transfer (WPT) with different methods.

Method	Number of Full-Wave Simulations		Overall CPU Time	
	$\eta_{\mathrm{LINK}}$	PTE	$\eta_{\mathrm{LINK}}$	PTE
gPC + macromodels	84	84	21 min 53 s	22 min
single gPC	495	495	2 h 4 min 35 s	2 h 4 min 42 s
Monte Carlo	10,000	10,000	41 h 48 min 25 s	41 h 50 min 46 s

## Abbreviations

The following abbreviations are used in this manuscript:

ISM	Industrial, Scientific and Medical
WPT	Wireless Power Transfer
PTE	Power Transfer Efficiency
gPC	generalized Polynomial Chaos
IoT	Internet of Things
RFID	Radio Frequency IDentification
RF	Radio Frequency
SCM	Stochastic Collocation Method
PDF	Probability Density Function
DC	Direct Current
DOA	Direction of Arrival
AC	Alternating Current
ADS	Advanced Design System
RV	Random Variables
ST	Stochastic Testing
SGH	Standard Gain Horn
CDF	Cumulative Distribution Function

## Appendix A. Stochastic Testing Point Selection

The selection of the *M* collocation points $x_{m}$ used in Equation (13), necessary to compute the coefficients $y_{k}$ in Equation (11), is carried out by means of the ST algorithm introduced in [36]. Thereto, we first construct the set of $R={\left(Q+1\right)}^{N}$ quadrature points $t_{r}$ by means of an *N*-dimensional tensor product Gaussian quadrature rule [39]. Then, this set of points is sorted according to their corresponding weight $w_{r}$, in a decreasing order, and the first point $t_{0}$ is selected as first collocation point $x_{0}$. Next, the following M×1 matrix V is computed:

(A1) $$\begin{array}{c} \begin{array}{c} V=\frac{\varphi (x_{0})}{∥\varphi \left(x_{0}\right)∥} \end{array} \end{array}$$

where $\varphi \left(x_{0}\right)={\left[\varphi_{0}\left(x_{0}\right),\ldots ,\varphi_{K}\left(x_{0}\right)\right]}^{T}$. Finally, all the other M−1 collocation points are selected by means of an iterative procedure. More specifically, a point $t_{r}$ is selected as a new collocation point if:

(A2) $$\begin{array}{c} \begin{array}{c} \frac{∥v\left(t_{r}\right)∥}{∥\varphi \left(t_{r}\right)∥}>\chi \end{array} \end{array}$$

where *χ* is a threshold number, in this manuscript selected to be $10^{-3}$, and $v(t_{r})$ is given by

(A3) $$\begin{array}{c} \begin{array}{c} v\left(t_{r}\right)=\varphi \left(t_{r}\right)-VV^{T}\varphi \left(t_{r}\right) \end{array} \end{array}$$

Then, the matrix V is updated by including the normalized vector:

(A4) $$\begin{array}{c} \begin{array}{c} \frac{v(t_{r})}{∥v\left(t_{r}\right)∥} \end{array} \end{array}$$

as a new column. The algorithm ends when *M* collocation points have been selected.
